# Distinguishing Obsessive-Compulsive Symptoms in Schizophrenia-Spectrum Disorders and Obsessive-Compulsive Disorder: The Role of Basic Self Disturbances

**DOI:** 10.1192/j.eurpsy.2025.2040

**Published:** 2025-08-26

**Authors:** V. Alves Barata, J. Bastos, T. Filipe Ferreira

**Affiliations:** 1 Hospital Prof. Doutor Fernando Fonseca, Lisbon, Portugal

## Abstract

**Introduction:**

Obsessive-compulsive symptoms (OCS) are frequently observed in both obsessive-compulsive disorder (OCD) and schizophrenia-spectrum disorders (SSD), creating significant diagnostic challenges. Historically, Karl Jaspers defined “true obsessions” as a struggle against intrusive ideas that appear nonsensical and “alien” to the personality, demarcating this concept from delusions and overvalued ideas, in which cases the person would be convinced of the relevance of the content. However, since the 1980s, the concepts of insight and resistance in OCD have been deemphasized in diagnostic criteria, broadening the definition of OCD to include cases with poor or absent insight. The broadening of these criteria has blurred the distinction between OCD and SSD and has narrowed the diagnosis of schizophrenia to primarily delusional and hallucinatory conditions, overlooking obsessive phenomena in this disorder.

**Objectives:**

The primary goal of this review is to differentiate the phenomenological features of OCS in OCD from those in SSD, focusing on the connection between obsessive-compulsive phenomena and disturbances in the basic self in SSD.

**Methods:**

A literature review was conducted using the keywords “obsessive-compulsive symptoms”; “schizophrenia”; “obsessive-compulsive disorder”; “phenomenology” in the Pubmed and Google Scholar databases.

**Results:**

The findings suggest that the underlying nature and subjective experience of OCS may differ substantially between OCD and SSD. An essential component of this differentiation is the exploration of basic self-disturbances, which refer to profound disruptions in an individual’s sense of ownership of experience and agency of action - elements often impacted in SSD but less so in OCD. Patients with SSD often experience OCS in a more alien and automatic manner, with intrusive thoughts and compulsions lacking a clear sense of personal ownership or agency. These obsessions are more likely to blend with delusional thinking and other psychotic features, reflecting broader disturbances in the basic self. The lack of insight and the feeling that obsessive thoughts are externally imposed or intruding from outside the self is a hallmark in these cases. As for compulsions, these may serve as maladaptive strategies to manage or compensate for self-disturbances, rather than purely to neutralize distress as seen in OCD.

**Conclusions:**

Accurate differentiation of OCS in SSD from those in OCD requires clinicians to focus on the quality of self-experience, particularly in terms of insight, ownership and agency. Recognizing how certain obsessive phenomena in SSD reflect disturbances in the basic self is crucial for improving diagnostic accuracy and ensuring appropriate treatment.

**Disclosure of Interest:**

None Declared

